# Private Medical Instruction

**Published:** 1842-09

**Authors:** 


PRIVATE MEDICAL INSTRUCTION.

We have received, and notice with pleasure, the circular of John R. W. Dunbar, M. D., late Professor of Surgery and Surgical Anatomy in Washington College, Baltimore, setting forth his arrangements for private tuition. They are more ample, diversified and judicious than any we have yet met with, and we sincerely wish him a numerous class. His terms are $50 for one winter, $100 for a year, and $200 for the whole term of pupilage. It is in vain to deny that the private tuition of medical students in the United States is, *on the main*, humbuggery. Respectable exceptions, we admit, are to be met with, but they are by no means common. It is this lamentable deficiency in our plans of medical education, that renders it impossible for the best regulated public schools to send forth such graduates as would do them honor and raise the character of our profession. We again say that Dr. Dunbar, and all who may do like him, have our best wishes. D.

